# Effects of traditional Chinese exercise on vascular function in patients with Alzheimer’s disease: A protocol for systematic review and network meta-analysis of randomized controlled trials

**DOI:** 10.1097/MD.0000000000032517

**Published:** 2023-01-20

**Authors:** Jin Li, Chen Wang, Peizhen Zhang

**Affiliations:** a School of Sports Medicine and Rehabilitation, Beijing Sport University, Beijing, China

**Keywords:** Alzheimer disease, network meta-analysis, protocol, traditional Chinese exercise, vascular function

## Abstract

Alzheimer’s disease (AD) is a progressive neurodegenerative disorder with an insidious onset, usually characterized by memory impairment, visual-spatial skill impairment, executive dysfunction and personality behavioral changes. Studies have confirmed that vascular dysfunction may precede AD pathological changes and can present as vascular malformations, atherosclerosis, and impaired self-regulation, and can affect oxidative stress and amyloidosis. Therefore, it is important to improve or prevent vascular dysfunction in AD patients. Regular exercise can effectively inhibit the production of reactive oxygen species during the occurrence of AD and can improve the reduction of cerebral blood flow due to AD. Previous studies have shown that exercise can achieve superior clinical results in improving vascular function in AD patients. Therefore, we hypothesize that traditional Chinese exercises (TCEs) may have a good clinical effect in improving vascular function in patients with AD.

**Methods:**

We will search “PubMed,” “the Cochrane Library,” “Embase,” “Web of Science,” “CINAHL,” “ProQuest Dissertations and Theses,” and “ProQuest-Health & Medical Collection,” “CNKI,” “SinoMed,” “VIP,” and “Wanfang Data” to find randomized controlled trials of the effects of TCEs on AD vascular function from the creation of the database to the present, including at least 1 indicator in carotid intima-media thickness (cIMT), middle cerebral artery mean flow velocity (MFV), blood indicators [Heme Oxidase-1 (HO-1), angiopoietin I (Ang I), vascular endothelial growth factor (VEGF), brain-derived neurotrophic factor, matrix metalloproteinase-9 (MMP-9)], and arterial stiffness [(Ankle Brachial Index (ABI), pulse wave velocity (PWV)]. For the included literature, Excel 2019 will be used for data extraction and collection. For the indicators that can be netted for network meta-analysis, Surface Under the Cumulative Ranking for each exercise modality will be calculated with the help of Stata 16.0 and rank, where the higher the SUCRA score, the higher the ranking. For the indicators that cannot be netted, Review Manager 5.4 will be used for meta-analysis will be performed to evaluate the improvement effect of TCEs on AD patients.

**Results:**

This meta-analysis will further determine the efficacy and safety of TCEs on vascular function in AD patients.

**Conclusion:**

In this study, randomized controlled trials of the effects of TCEs on vascular function in AD patients will be selected to provide evidence-based medical evidence for promoting the application of TCEs by observing the order of advantages and disadvantages of various exercise modalities through network meta-analysis.

## 1. Introduction

Alzheimer’s disease (AD) is a progressive brain disease with a specific onset of age-related cognitive and functional decline leading to death^[1]^ characterized by pathology of excessive accumulation of amyloid-β (Aβ) and tau protein, neuronal and vascular damage, reduced cerebral glucose metabolism^.[2,3]^ Its symptoms are often characterized by significant memory loss and deficits in executive function, visuospatial ability, and attention.^[4]^

Several recent studies have explored the different mechanisms of neurotic plaque deposition that may be involved in the pathogenesis and development of AD.^[5–8]^ One study reported vascular alterations in 50% of AD patients, but the role of age-related vascular factors in the pathogenesis of AD is still not fully understood.^[8]^ The reported changes in vascular function in patients with AD are cerebral capillary shrinkage, focal contraction, decreased temporal and frontal cortical perfusion, structural changes in the endothelium, and deposition of Aβ in the vascular wall.^[9]^ Based on these factors,^[6,9,10]^ we assume that the main sites affected by Aβ deposition and AD onset are concentrated in the central nervous system (blood vessels, small arteries, arterioles and capillaries). Vascular dysfunction in AD patients may be manifested throughout the body. In particular, nitric oxide (NO) bio-availability is reduced and blood flow is decreased, independently of vascular risk factors. Cerebrovascular, extracranial vascular, and peripheral vascular dysfunction may be linearly related to cognitive dysfunction, indicating that central and peripheral vascular function may have an important function in AD patients.^[11,12]^

Much evidence suggests that vascular dysfunction may precede the onset of classical AD pathology,^[13]^ and the risk of AD may rise with increased vascular risk factors.^[14]^ Therefore, improving or delaying the development of vascular dysfunction is important for the occurrence and progression of AD. Currently, there are no effective drugs to slow the development of AD in humans,^[15]^ and the best way to intervene is to slow the onset of symptoms before they develop and promote early intervention. Even though most AD cannot be improved by lifestyle interventions, it is important to do so to modify risk factors.^[16]^

Exercise is well-known to be one of the most effective nonmedical means of vascular protection and has a strong impact on vascular function.^[17,18]^ Indeed, exercise has a positive direct effect on blood flow (BF), vascular reactivity, vascular endothelial growth factor (VEGF) and angiogenesis, upregulating NO bioavailability and growth factor expression.^[19,20]^ There is growing interest in the beneficial effects of exercise on vascular and cognitive function in patients with AD. However, the specific mechanisms of how exercise positively affects adaptations of peripheral vascular in AD patients remain controversial.^[21–24]^ Pedrinolla et al showed that 45 minutes of walking training improved AD patients’ peripheral vessel function. It is possible that VEGF and NO levels are up-regulated due to the repetitive increase in shear rate during exercise.^[25]^

TCEs are based on the doctrine of “yin and yang” and “the holistic concept” as the guiding ideology, and through mobilization of the body’s own potential, 1 can achieve strengthening the body, cure and prevent diseases, prevent and improve health. TCEs usually include Taijiquan (TJQ), Baduanjin (BDJ), Wuqinxi (WQX), Liuzijue Qigong (LQG), and Yijinjing (YJJ).^[26]^ All of these exercises must be performed in conjunction with breathing in order to tone the body, regulate the breath, and improve the health and function of the body. Recent researches have demonstrated that TCEs can increase the quality of life, cardiopulmonary endurance, and vascular function in patients with coronary heart disease.^[27–30]^ It has also been shown that TCEs can effectively improve the level of total cholesterol,^[31]^ triglyceride, high-density lipoprotein (HDL), and low-density lipoprotein in patients with atherosclerosis.^[32–35]^

The mechanisms involved in the effects of TCEs on vascular function are listed as follows: TJQ takes the waist as the axis and drives the limbs, causing muscles to alternately contract and relax regularly, adjusting the body’s yin and yang, unblocking the meridians, and harmonizing the qi and blood. It not only increases the number of capillaries, improves the elasticity of blood vessels, reduces peripheral resistance, and prevents the development of atherosclerosis, but also increases blood volume, reduces blood viscosity and blood lipids, and thus improves the function of blood vessels.^[36,37]^ BDJ can promote the flow of qi and blood throughout the body and enhance the body’s ability to resist external evil, and positively affects vascular function by increasing vascular elasticity and lowering blood pressure to improve vascular endothelial function, which in turn enhances the efficiency of oxygen transportation and utilization in the body’s blood circulation.^[38,39]^ By imitating the different movements of different animals, WQX stimulates the meridians and acupuncture points, improves the blood supply function of the heart, accelerates the circulation of blood around the body, opens the meridians and channels, unblocks the blood and qi, and strengthens the body.^[40,41]^ It reduces the overexcited sympathetic nerves, lowers blood pressure, and reduces endothelial inflammatory responses.^[42,43]^ LQG can promote the movement of qi and blood in the body, regulate the functions of the internal organs, unblock the meridians, qi, and blood, harmonize breathing, promote blood circulation, enhance lipase activity, increase blood lipid metabolism, and improve vascular function.^[44–46]^ YJJ has a beneficial effect on vascular function by pulling on the meridians, regulating the functions of the internal organs, promoting blood circulation, enhancing vascular compliance, lowering blood pressure, improving blood lipid levels, and preventing or slowing down atherosclerosis.^[47–49]^

Based on the above studies, we found that TCEs have significant advantages in improving cardiovascular function. TCEs (specifically TJQ, BDJ, LQG, WXQ, and YJJ) have significant advantages in preventing and delaying cardiovascular diseases. Some studies have confirmed that their moderate exercise intensity and safe intervention process can reduce blood pressure, blood lipids, and other cardiovascular disease risk factors, and can increase exercise capacity, improve quality of life, and balance physical and mental health. Compared with other forms of exercise, TCEs have the advantages of moderate and safe exercise intensity, less stimulation of the cardiovascular system, and no restrictions on the venue.^[50]^

We hypothesized that TCEs could improve vascular function in AD patients. However, the number of relevant studies is currently insufficient. Therefore, the aim of this study is to select 5 TCEs as the main interventions and compare them directly or indirectly by network meta-analysis (NMA) to observe which TCE has the best effect on improving vascular function in AD patients, in order to lay the foundation for the promotion of TCEs in clinical practice and community.

## 2. Methods and analysis

**Figure 1.**
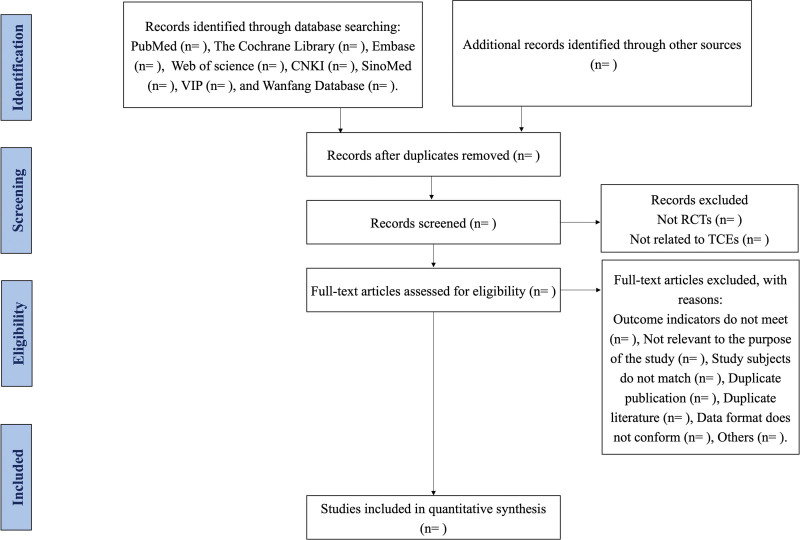
PRISMA diagram of the study selection process.

This systematic review and network meta-analysis of randomized controlled trials follows the Preferred Reporting Items for Systematic Reviews and Meta-Analyses Protocols (PRISMA-P) statement^[51]^ (Fig. 1), which have been registered in advance in the International prospective register of systematic reviews (PROSPERO) (registration number: CRD42022303349).

## 3. Search strategy

We will search 7 English databases “PubMed,” “the Cochrane Library,” “Embase,” “Web of Science,” “CINAHL,” “ProQuest Dissertations and Theses,” and “ProQuest-Health & Medical Collection,” as well as 4 Chinese databases: “CNKI,” “SinoMed,” “VIP,” and “Wanfang Data,” from the date the database was created to the present. Manual searches will be conducted on sites such as Baidu Xueshu and Google Scholar to add literature that may be missing or insufficient. In the process of searching for articles, additional literature will be included in this study at the same time if appropriate references are found when reading the articles.

In each database, we will search using the following search terms: “taijiquan, tai chi, taiji, taiqi, tai chi chuan, tai chi quan, tai chi qigong, shadow boxing, qi gong, baduanjin, 8 section brocades, wuqinxi, 5-animal exercises, 5-animal boxing, yijinjing, liuzijue, 6-character formula, 6-word qigong, traditional Chinese exercise (TCE), Chinese traditional sports” and “Alzheimer disease, Alzheimer disease, Alzheimer, Alzheimer, dementia, senile dementia, aged dementia, feeble-mindedness, dementia aphrenia, elderly, aged” and “vascular function, vessel function, blood vessel function, cardiovascular function, carotid intima-media thickness (cIMT), middle cerebral artery mean flow velocity (MFV), blood indicators, heme oxidase-1, angiopoietin I, vascular endothelial growth factor, brain derived neurotrophic factor, matrix metalloproteinase-9 (MMP-9), arterial stiffness, ankle brachial index, pulse wave velocity.” The search strategy for PubMed is shown in Table 1 as an example. The detailed search strategy is fully described in Supplemental material 2, http://links.lww.com/MD/I309.

**Table 1:** Search strategy to be used in PubMed.

Number	Search terms
1	taijiquan or tai chi or taiji or taiqi or tai chi chuan or tai chi quan or tai chi qigong or shadow boxing or qigong
2	baduanjin or 8 section brocades
3	wuqinxi or 5-animal exercises or 5-animal boxing
4	yijinjing
5	liuzijue or 6-character formula or 6-word qigong
6	traditional Chinese exercise or Chinese Traditional Sports
7	1 or 2 or 3 or 4 or 5 or 6
8	Alzheimer disease or Alzheimer disease or Alzheimer or Alzheimer
9	dementia or senile dementia or aged dementia or feeble-mindedness or dementia aphrenia or elderly or aged
10	8 or 9
11	vascular function or vessel function or blood vessel function or cardiovascular function
12	carotid intima-media thickness
13	middle cerebral artery mean flow velocity
14	blood indicators or Heme Oxidase-1 or Angiopoietin I or Vascular Endothelial Growth Factor or Brain Derived Neurotrophic Factor or Matrix metalloproteinase-9
15	arterial stiffness
16	Ankle Brachial Index
17	Pulse wave velocity
18	11 or 12 or 13 or 14 or 15 or 16 or 17
19	7 and 10 and 18

We will identify and include randomized controlled trials (RCT) on the effects of TCEs on at least 1 vascular function in cIMT, middle cerebral artery MFV, blood indicators (HO-1, Ang I, VEGF, brain-derived neurotrophic factor (BDNF), MMP-9), and arterial stiffness (ABI, PWV).

## 4. Eligibility criteria

Inclusion criteria:

1. Type of study: RCT of TCEs on vascular function in AD.
2. Subjects: people with AD, with no restrictions on gender or nationality.
3. Intervention measures: TCEs (specifically TJQ, BDJ, WQX, LQG, and YJJ).
4. Control group: usual care or only took medicine group.
5. Outcome: including at least 1 indicator in cIMT, middle cerebral artery MFV, blood indicators (HO-1, Ang I, VEGF, BDNF, MMP-9), and arterial stiffness (ABI, PWV).

Exclusion criteria:

1. Non-RCTs: such as review, clinical experience summary literature, conferences, etc
2. The study subjects, exercise mode, and outcome indicators do not meet the inclusion criteria or were not related to the study purpose.
3. Repeated publication and duplication of literature.
4. Incomplete literature data, data format that does not conform, data that cannot be converted, etc
5. Retracted literature.

## 5. Data collection and analysis

### 5.1. Study selection

We will import the literature retrieved in each database into EndNote X20 and remove duplicates. Two reviewers (JL, CW) will read the titles and abstracts of the literature to remove irrelevant literature according to the inclusion and exclusion criteria independently. If the reading of the title and abstract do not determine whether the study is to be included, the decision will be made by reading the whole article. If any disagreements occur, literature screening process will be negotiated with a third researcher (PZZ) through a group discussion. We will use a PRISMA diagram to illustrate the entire process of selecting literature for screening.

### 5.2. Data extraction and collection

By reading the full text of the final included literature, relevant information will be extracted and recorded using Excel 2019, including: ① General information about the literature: first author, country/region, and year published. ② Basic details of study subjects (recorded according to different groups): age, gender, and sample size. ③Intervention measures (recorded according to different groups): exercise mode, exercise frequency, and duration of each exercise. ④ Outcome indicators: cIMT, middle cerebral artery MFV, blood indicators (HO-1, Ang I, VEGF, BDNF, MMP-9), and arterial stiffness (ABI, PWV). In case of uncertainty or disagreement, an attempt will be made to contact the study authors via email for details. If there are missing data in the included original studies, the missing original data will be obtained from the first author or corresponding author.

### 5.3. Dealing with missing data

Because the outcome indicators of this study will be continuous variables, if data from included studies are shown as medians, confidence intervals, or *P*-values, we attempt to convert them to the form of means and standard deviations by calculation. During data extraction, if insufficient or missing data are found in the literature, we will attempt to contact the authors via email to obtain complete data. In case of unsuccessful contact or if the authors are unable to provide the original data and the data results can not be converted into the form of mean and standard deviation, we will decide whether to include the literature through group discussion regarding the specific situation.

### 5.4. Risk of quality assessments of included trials

Two reviewers will use the Cochrane Collaboration’s tool for assessing risk of bias in randomized trials to evaluate the quality of the final included studies in 7 areas (random sequence generation, allocation concealment, blinding of patients and personnel, blinding of outcome assessors, incomplete outcome data, selective reporting, and other biases).^[52]^ The results of each determination will be categorized into 3 levels of Low Risk, High Risk, and Unclear. Reasons will be given for the rating of Unclear. Any inconsistency in the evaluation results will be resolved by discussion with a third reviewer. The relevant evaluation results are entered into Review Manager 5.4, and the relevant graphs will generate and analyze.

## 6. Statistical analyses

The outcome indicators in this study are continuous variables, and the means and standard deviations of the outcome indicators in the included literature will be extracted for data analysis. If the outcome indicators in the included literature are presented in the form of non-mean and standard deviation, they will be transformed into the form of mean and standard deviation by arithmetic for this study. In this study, we will select cIMT, middle cerebral artery MFV, blood indicators (HO-1, Ang I, VEGF, BDNF, MMP-9), and arterial stiffness (ABI, PWV), all of which will be statistically analyzed for the change in difference before and after the intervention.

Stata 16.0 software (College Station, Texas 77845 USA) will be used to perform network meta-analysis and funnel plotting to compare and rank different TCEs. The inconsistency model test will be first performed, and if *P* > .05, the inconsistency model test will not be significant, and use the consistency model. The node splitting method will be used to test for local inconsistency. For the results, the Surface Under the Cumulative Ranking of each intervention will be calculated for ranking each intervention,^[53]^ where the larger the Surface Under the Cumulative Ranking value, the better the ranking.^[54]^ The ranking of different TCE modalities for different indicators will be obtained.

If obtaining outcome indicators for NMA are not possible, traditional meta-analysis will be performed with the help of Review Manager 5.4. Based on the *I*^2^ value, the appropriate model will be selected. If *I*^2^ > 50%, a random effects model (RE) analysis and a fixed effects model (FE) analysis will be selected for *I*^2^ ≤ 50%. Funnel plots will be drawn and generated to detect the possibility of reporting bias in the included studies.

Egger test will be used to detect whether there is a significant publication bias in the included studies and whether the Meta-analysis results are stable. If *P* < .05 and 95% confidence interval does not contain 0, then publication bias exists and Meta-analysis results will be unstable.

## 7. Subgroup analysis

When sufficient data will be included in the study, we will use subgroup analysis. Subgroup analysis will be performed according to age, gender, duration of intervention, time since AD onset (take a decade as a stage), and type of control group (medication, no additional TCEs, and exercise with non-TCEs), etc The analysis of intervention effects will be performed using the χ^2^ test, with *P* < .05 indicating a statistically significant difference between subgroups.

### 7.1. Sensitivity analysis

Sensitivity analysis will be performed to exclude experiments with small sample sizes (i.e., a group of <10 subjects) and to exclude studies with a high risk of bias (e.g., the sequence generation method is nonrandomized, etc) to assess the stability of the results.

## 8. Patient and public involvement

This study will conduct without patient or public participation. It is a summary and analysis of previous studies.

## 9. Possible corrections

Currently, we do not expect to make any modifications to this study protocol during the course of the study to avoid bias in the results. Nevertheless, after discussion if necessary modifications occur, they will be made on the website (PROSPERO) where this study protocol is registered (reference number: CRD42022303349).

## 10. Recommendations assessment

GRADE will be used to grade the quality of the evidence and the degree of recommendation. The evidence will be graded as high, moderate, low, and very low according to GRADE that take into account study design type, risk of bias, publication bias, effect size, dose response, and other confounding factors. All researchers will evaluate independently, and disagreements, if any, will be resolved through group discussions.

## 11. Discussion

Patients with AD typically exhibit impaired brain health and function, and 50% of AD patients have vascular alterations.^[8]^ AD risk increases with the augment of vascular risk factors.^[14]^ Vascular dysfunction in AD typically manifests by impacting amyloid pathways, reducing Aβ clearance, increasing Aβ production, elevating Aβ levels in the brain^[55]^ disrupting the blood-brain barrier, hypoperfusion-hypoxia, impairing the endothelial metabolism,^[56]^ causing small artery and capillary malformations, increasing vascular curvature,^[57]^ impairing self-regulation, and reducing vasodilatory response.^[58]^ Exercise can promote cerebrovascular health and function, maintain normal neurogenesis, neurotransmission, and synaptic plasticity, improve vascular morphology and function, increase Aβ clearance, and can have multiple effects on AD-related vascular factors (e.g., VEGF, BDNF.).^[59]^ A study by Theodorou in patients with coronary artery disease (CHD) indicated that aerobic exercise had a beneficial effect on lipid metabolism (triglyceride, TC, HDL-C, low-density lipoprotein-C).^[60]^ Donley study in patients with metabolic syndrome showed that aerobic exercise reduced their arterial stiffness by decreasing carotid-femoral pulse wave velocity (cfPWV, cIMT).^[61]^

We also found the following clinical effects of TCEs in studies related to the improvement of vascular function: TJQ can effectively improve carotid atherosclerosis, which may be associated with enhanced aerobic metabolism, serum NO, HDL, and the HDL/TC ratio in body. It could regulate cytokine and lipid metabolism and protect vascular endothelial function, improve vascular compliance, and lower blood pressure.^[62,63]^ BDJ may improve vascular endothelial cell disorders by lowering blood pressure, maintaining normal blood flow, inhibiting cytokine secretion, and protecting vascular endothelial cells.^[64]^ WQX can effectively improve vascular elastic condition, increase blood volume, improve blood concentration and flow velocity, help to exclude blood circulation disorders and small-vessel spasm, and promote venous blood return.^[65]^ Long-term performance of LQG can improve endothelium-dependent diastolic function and affects lipid metabolism in vascular endothelial cells, thereby reducing lipid levels and improving cardiovascular system function.^[66]^ YJJ can effectively promote blood circulation and improve metabolic levels, improve vascular elasticity, reduce cardiac afterload, and lower blood pressure.^[67]^ The above TCE studies confirm their clinical effectiveness in improving lipid metabolism and vascular stiffness.

Currently, there are no Meta-analyses to assess the effects of TCEs on vascular function in AD. Therefore, in this study, we comprehensively assessed the effects of TCEs on vascular function [cIMT, middle cerebral artery MFV, blood indicators (HO-1, Ang I, VEGF, BDNF, MMP-9), and arterial stiffness (ABI, PWV)] in AD. Our goals were to explore TCEs that are compatible with the disease progression of AD patients through NMA, to provide professional evidence that different TCEs can improve their vascular function of AD patients, and to personalize the selection of exercise modalities to improve vascular function and maximize the effects of TCEs according to the patient’s condition.

## Acknowledgments

Not applicable.

## Author contributions

**Conceptualization:** Jin Li, Chen Wang, Peizhen Zhang.

**Formal analysis:** Jin Li.

**Funding acquisition:** Peizhen Zhang.

**Investigation:** Chen Wang.

**Methodology:** Jin Li.

**Project administration:** Jin Li.

**Supervision:** Peizhen Zhang.

**Validation:** Peizhen Zhang.

**Writing—original draft:** Jin Li, Chen Wang, Peizhen Zhang.

**Writing—review and editing:** Peizhen Zhang.

## Supplementary Material
