# Supplementary material for: Effects of traditional Chinese exercise on vascular function in patients with Alzheimer’s disease: A protocol for systematic review and network meta-analysis of randomized controlled trials
Source: Medicine (Baltimore). 2023 Jan 20;102(3):e32517. doi: 10.1097/MD.0000000000032517 (PMC9857473; doi:10.1097/MD.0000000000032517)
Supplement: Supplementary file 1 [file medi-102-e32517-s001.pdf]

Summary of search formulas for each database

1. PubMed

#1: ((((((((taijiquan) OR (tai chi)) OR (taiji)) OR (taiqi)) OR (tai chi chuan)) OR (tai chi quan)) OR (tai chi qigong)) OR (shadow boxing)) OR (qigong)

#2: (baduanjin) OR (eight section brocades)

#3: (((wuqinxi)) OR (five-animal exercises)) OR (five-animal boxing)

#4: yijinjing

#5: (((liuzijue)) OR (six-character formula)) OR (six-word qigong)

#6: (traditional Chinese exercise) OR (Chinese traditional sports)

#7: #1 OR #2 OR #3 OR #4 OR #5 OR #6

#8: (((((Alzheimer's disease) OR (Alzheimer disease)) OR (Alzheimer)) OR (Alzheimer's)

#9: (((((((dementia)) OR (senile dementia)) OR (aged dementia)) OR (feeble-mindedness)) OR (dementia aphrenia)) OR (elderly)) OR (aged)

#10: #8 OR #9

#11: (((((vascular function) OR (vessel function)) OR (blood vessel function)) OR (cardiovascular function)

#12: carotid intima-media thickness

#13: middle cerebral artery mean flow velocity

#14: ((((((((((blood indicators)) OR (heme oxidase-1)) OR (angiopoietin I)) OR (vascular endothelial growth factor)) OR (brain derived neurotrophic factor)) OR (matrix metalloproteinase-9)

#15: arterial stiffness

#16: ankle brachial index

#17: pulse wave velocity

#18: #11 OR #12 OR #13 OR #14 OR #15 OR #16 OR #17

#19: #7 AND #10 AND #18

2. the Cochrane Library

#1: (taijiquan) OR (tai chi) OR (taiji) OR (taiqi) OR (tai chi chuan) OR (tai chi quan) OR (tai chi qigong) OR (shadow boxing) OR (qigong) OR (baduanjin) OR (eight section brocades) OR (wuqinxi) OR (five-animal exercises) OR (five-animal boxing) OR (yijinjing) OR (liuzijue) OR (six-character formula) OR (six-word qigong) OR (traditional Chinese exercise) OR (Chinese traditional sports)

#2: (Alzheimers disease) OR (Alzheimer disease) OR (Alzheimer) OR (Alzheimers) OR (dementia) OR (senile dementia) OR (aged dementia) OR (feeble-mindedness) OR (dementia aphrenia) OR (elderly) OR (aged)

#3: (vascular function) OR (vessel function) OR (blood vessel function) OR (cardiovascular function) OR (carotid intima-media thickness) OR (middle cerebral artery mean flow velocity) OR (blood indicators) OR (heme oxidase-1) OR (angiopoietin I) OR (vascular endothelial growth factor) OR (brain derived neurotrophic factor) OR (matrix metalloproteinase-9) OR (arterial stiffness) OR (ankle brachial index) OR (pulse wave velocity)

#4: #1 AND #2 AND #3

3. Embase

#1: 'taijiquan'/exp OR taijiquan OR 'tai chi'/exp OR 'tai chi' OR (tai AND chi) OR taiji OR taiqi OR 'tai chi chuan'/exp OR 'tai chi chuan' OR (tai AND chi AND chuan) OR 'tai chi quan' OR (tai AND chi AND quan) OR 'tai chi qigong' OR (tai AND chi AND ('qigong'/exp OR qigong)) OR 'shadow boxing' OR (('shadow'/exp OR shadow) AND ('boxing'/exp OR boxing)) OR 'baduanjin'/exp OR baduanjin OR 'eight section brocades' OR (eight AND ('section'/exp OR section) AND brocades) OR wuqinxi OR 'five-animal exercises' OR ('five animal' AND exercises) OR 'five-animal boxing' OR ('five animal' AND ('boxing'/exp OR boxing)) OR yijinjing OR liuzijue OR 'six-character formula' OR ('six character' AND ('formula'/exp OR formula)) OR 'six-word qigong' OR ('six word' AND ('qigong'/exp OR qigong)) OR 'traditional chinese exercise' OR (traditional AND ('chinese'/exp OR chinese) AND ('exercise'/exp OR exercise)) OR 'chinese traditional sports' OR (('chinese'/exp OR chinese) AND

traditional AND ('sports'/exp OR sports))

#2: alzheimers AND ('disease'/exp OR disease) OR (alzheimer AND ('disease'/exp OR disease)) OR alzheimer OR alzheimers OR 'dementia'/exp OR dementia OR (senile AND ('dementia'/exp OR dementia)) OR (('aged'/exp OR aged) AND ('dementia'/exp OR dementia)) OR 'feeble mindedness' OR (('dementia'/exp OR dementia) AND aphrenia) OR 'elderly'/exp OR elderly OR 'aged'/exp OR aged

#3: 'vascular function'/exp OR 'vascular function' OR (vascular AND ('function'/exp OR function)) OR 'vessel function' OR (('vessel'/exp OR vessel) AND ('function'/exp OR function)) OR 'blood vessel function'/exp OR 'blood vessel function' OR (('blood'/exp OR blood) AND ('vessel'/exp OR vessel) AND ('function'/exp OR function)) OR 'cardiovascular function'/exp OR 'cardiovascular function' OR (('cardiovascular'/exp OR cardiovascular) AND ('function'/exp OR function)) OR 'carotid intima-media thickness'/exp OR 'carotid intima-media thickness' OR (('carotid'/exp OR carotid) AND 'intima media' AND ('thickness'/exp OR thickness)) OR 'middle cerebral artery mean flow velocity' OR (middle AND cerebral AND ('artery'/exp OR artery) AND ('mean'/exp OR mean) AND ('flow'/exp OR flow) AND ('velocity'/exp OR velocity)) OR 'blood indicators' OR (('blood'/exp OR blood) AND ('indicators'/exp OR indicators)) OR 'heme oxidase-1' OR (('heme'/exp OR heme) AND 'oxidase 1') OR 'angiopoietin i' OR (('angiopoietin'/exp OR angiopoietin) AND i) OR 'vascular endothelial growth factor'/exp OR 'vascular endothelial growth factor' OR (vascular AND endothelial AND ('growth'/exp OR growth) AND factor) OR 'brain derived neurotrophic factor'/exp OR 'brain derived neurotrophic factor' OR (('brain'/exp OR brain) AND derived AND neurotrophic AND factor) OR 'matrix metalloproteinase-9'/exp OR 'matrix metalloproteinase-9' OR (('matrix'/exp OR matrix) AND ('metalloproteinase 9'/exp OR 'metalloproteinase 9')) OR 'arterial stiffness'/exp OR 'arterial stiffness' OR (arterial AND ('stiffness'/exp OR stiffness)) OR 'ankle brachial index'/exp OR 'ankle brachial index' OR (('ankle'/exp OR ankle) AND brachial AND ('index'/exp OR index)) OR 'pulse wave velocity'/exp OR 'pulse wave velocity' OR (('pulse'/exp OR pulse) AND ('wave'/exp OR wave) AND ('velocity'/exp OR velocity))

#4: #1 AND #2 AND #3

4. Web of Science

#1: TS=((taijiquan) OR (tai chi) OR (taiji) OR (taiqi) OR (tai chi chuan) OR (tai chi quan) OR (tai chi qigong) OR (shadow boxing) OR (qigong) OR (baduanjin) OR (eight section brocades) OR (wuqinxi) OR (five-animal exercises) OR (five-animal boxing) OR (yijinjing) OR (liuzijue) OR (six-character formula) OR (six-word qigong) OR (traditional Chinese exercise) OR (Chinese traditional sports)

#2: TS=((Alzheimer's disease) OR (Alzheimer disease) OR (Alzheimer) OR (Alzheimer's) OR (dementia) OR (senile dementia) OR (aged dementia) OR (feeble-mindedness) OR (dementia aphrenia) OR (elderly) OR (aged)))

#3: TS=((vascular function) OR (vessel function) OR (blood vessel function) OR (cardiovascular function) OR (carotid intima-media thickness) OR (middle cerebral artery mean flow velocity) OR (blood indicators) OR (heme oxidase-1) OR (angiopoietin I) OR (vascular endothelial growth factor) OR (brain derived neurotrophic factor) OR (matrix metalloproteinase-9) OR (arterial stiffness) OR (ankle brachial index) OR (pulse wave velocity))

#4: #1 AND #2 AND #3

5. CINAHL

#1: (taijiquan) OR (tai chi) OR (taiji) OR (taiqi) OR (tai chi chuan) OR (tai chi quan) OR (tai chi qigong) OR (shadow boxing) OR (qigong) OR (baduanjin) OR (eight section brocades) OR (wuqinxi) OR (five-animal exercises) OR (five-animal boxing) OR (yijinjing) OR (liuzijue) OR (six-character formula) OR (six-word qigong) OR (traditional Chinese exercise) OR (Chinese traditional sports)

#2: (Alzheimer's disease) OR (Alzheimer disease) OR (Alzheimer) OR (Alzheimer's) OR (dementia) OR (senile dementia) OR (aged dementia) OR (feeble-mindedness) OR (dementia aphrenia) OR (elderly) OR (aged)

#3: (vascular function) OR (vessel function) OR (blood vessel function) OR (cardiovascular function) OR (carotid intima-media thickness) OR (middle cerebral artery mean flow velocity) OR (blood indicators) OR (heme oxidase-1) OR

(angiopoietin I) OR (vascular endothelial growth factor) OR (brain derived neurotrophic factor) OR (matrix metalloproteinase-9) OR (arterial stiffness) OR (ankle brachial index) OR (pulse wave velocity)

#4: #1 AND #2 AND #3

6. ProQuest Dissertations and Theses

#1: (taijiquan) OR (tai chi) OR (taiji) OR (taiqi) OR (tai chi chuan) OR (tai chi quan) OR (tai chi qigong) OR (shadow boxing) OR (qigong) OR (baduanjin) OR (eight section brocades) OR (wuqinxi) OR (five-animal exercises) OR (five-animal boxing) OR (yijinjing) OR (liuzijue) OR (six-character formula) OR (six-word qigong) OR (traditional Chinese exercise) OR (Chinese traditional sports)

#2: (Alzheimer's disease) OR (Alzheimer disease) OR (Alzheimer) OR (Alzheimer's) OR (dementia) OR (senile dementia) OR (aged dementia) OR (feeble-mindedness) OR (dementia aphrenia) OR (elderly) OR (aged)

#3: (vascular function) OR (vessel function) OR (blood vessel function) OR (cardiovascular function) OR (carotid intima-media thickness) OR (middle cerebral artery mean flow velocity) OR (blood indicators) OR (heme oxidase-1) OR (angiopoietin I) OR (vascular endothelial growth factor) OR (brain derived neurotrophic factor) OR (matrix metalloproteinase-9) OR (arterial stiffness) OR (ankle brachial index) OR (pulse wave velocity)

#4: #1 AND #2 AND #3

7. ProQuest-Health & Medical Collection

#1: (taijiquan) OR (tai chi) OR (taiji) OR (taiqi) OR (tai chi chuan) OR (tai chi quan) OR (tai chi qigong) OR (shadow boxing) OR (qigong) OR (baduanjin) OR (eight section brocades) OR (wuqinxi) OR (five-animal exercises) OR (five-animal boxing) OR (yijinjing) OR (liuzijue) OR (six-character formula) OR (six-word qigong) OR (traditional Chinese exercise) OR (Chinese traditional sports)

#2: (Alzheimer's disease) OR (Alzheimer disease) OR (Alzheimer) OR (Alzheimer's) OR (dementia) OR (senile dementia) OR (aged dementia) OR (feeble-mindedness) OR (dementia aphrenia) OR (elderly) OR (aged)

#3: (vascular function) OR (vessel function) OR (blood vessel function) OR (cardiovascular function) OR (carotid intima-media thickness) OR (middle cerebral artery mean flow velocity) OR (blood indicators) OR (heme oxidase-1) OR (angiopoietin I) OR (vascular endothelial growth factor) OR (brain derived neurotrophic factor) OR (matrix metalloproteinase-9) OR (arterial stiffness) OR (ankle brachial index) OR (pulse wave velocity)

#4: #1 AND #2 AND #3

8. CNKI

#1: (主题=太极拳) OR (主题=太极) OR (主题=太极气功) OR (主题=八段锦) OR (主题=六字诀) OR (主题=易筋经) OR (主题=五禽戏) OR (主题=传统运动) OR (主题=传统疗法)

#2: (主题=阿尔兹海默症) OR (主题=阿尔茨海默病) OR (主题=阿尔茨海默症) OR (主题=阿尔兹海默病) OR (主题=痴呆) OR (主题=老年性痴呆) OR (主题=老年)

#3: (主题=血管功能) OR (主题=血管) OR (主题=心血管) OR (主题=颈动脉内膜中层厚度) OR (主题=大脑中动脉平均流速) OR (主题=血液指标) OR (主题= heme oxidase-1) OR (主题= angiopoietin I) OR (主题=血管内皮生长因子) OR (主题=脑源性神经营养因子) OR (主题= matrix metalloproteinase-9) OR (主题=动脉硬度) OR (主题=动脉僵硬度) OR (主题=踝肱指数) OR (主题=脉搏波速度)

#4: #1 AND #2 AND #3

9. SinoMed

#1: "太极拳"[全部字段:智能] OR "太极"[全部字段:智能] OR "太极气功"[全部字段:智能] OR "八段锦"[全部字段:智能] OR "六字诀"[全部字段:智能] OR "易筋经"[全部字段:智能] OR "五禽戏"[全部字段:智能] OR "传统运动"[全部字段:智能] OR "传统疗法"[全部字段:智能]

#2: "阿尔兹海默症"[全部字段:智能] OR "阿尔茨海默病"[常用字段:智能] OR "阿尔茨海默症"[常用字段:智能] OR "阿尔兹海默病"[常用字段:智能] OR "痴呆"[常用字段:智能] OR "老年性痴呆"[常用字段:智能] OR "老年"[常用字段:智能]

#3: "血管"[全部字段:智能] OR "心血管"[全部字段:智能] OR "血管功能"[全部字段:智能] OR "颈动脉内膜中层厚度"[全部字段:智能] OR "大脑中动脉平均流速"[全部字段:智能] OR "血液指标"[全部字段:智能] OR "heme"[全部字段:智能] AND "oxidase-1"[全部字段:智能] OR "angiopoietin"[全部字段:智能] AND "I"[全部字段:智能] OR "血管内皮生长因子"[全部字段:智能] OR "脑源性神经营养因子"[全部字段:智能] OR "matrix"[全部字段:智能] AND "metalloproteinase-9"[全部字段:智能] OR "动脉硬度"[常用字段:智能] OR "动脉僵硬度"[常用字段:智能] OR "踝肱指数"[常用字段:智能] OR "脉搏波速度"[常用字段:智能]

#4: #1 AND #2 AND #3

10. VIP

#1: ((((任意字段=太极拳 OR 任意字段=太极) OR 任意字段=太极气功) OR 任意字段=八段锦) OR 任意字段=六字诀)

#2: ((((任意字段=易筋经 OR 任意字段=五禽戏) OR 任意字段=传统运动) OR 任意字段=传统疗法)

#3: #1 OR #2

#4: (((((((任意字段=阿尔兹海默症 OR 任意字段=阿尔茨海默病) OR 任意字段=阿尔茨海默症) OR 任意字段=阿尔兹海默病) OR 任意字段=痴呆) OR 任意字段=痴呆症) OR 任意字段=老年性痴呆) OR 任意字段=老年)

#5: ((((任意字段=太极拳 OR 任意字段=太极) OR 任意字段=太极气功) OR 任意字段=八段锦) OR 任意字段=六字诀)

#6: ((((任意字段=血管 OR 任意字段=心血管) OR 任意字段=血管功能) OR 任意字段=颈动脉内膜中层厚度) OR 任意字段=大脑中动脉平均流速)

#7: ((((任意字段=血液指标 OR 任意字段= heme oxidase-1) OR 任意字段= angiopoietin I) OR 任意字段=血管内皮生长因子) OR 任意字段=脑源性神经营养因子)

#8: ((((任意字段= matrix metalloproteinase-9 OR 任意字段= 动脉硬度) OR 任意字段= 动脉僵硬度) OR 任意字段=踝肱指数) OR 任意字段=脉搏波速度)

#9: #5 OR #6 OR #7 OR #8

#10: #3 AND #4 AND #9

11. Wanfang Data

#1: 全部:(太极拳) or 全部:(太极) or 全部:(太极气功) or 全部:(八段锦) or 全部:(六字诀) or 全部:(易筋经) or 全部:(五禽戏) or 全部:(传统运动) or 全部:(传统疗法)

#2: 全部:(阿尔兹海默症) or 全部:(阿尔茨海默病) or 全部:(阿尔茨海默症) or 全部:(阿尔兹海默病) 全部:(痴呆) or 全部:(老年性痴呆) or 全部:(老年)

#3: 全部:(血管) or 全部:(心血管) or 全部:(血管功能) or 全部:(颈动脉内膜中层厚度) or 全部:(大脑中动脉平均流速) or 全部:(血液指标) or 全部:(heme oxidase-1) or 全部:(angiopoietin I) or 全部:(血管内皮生长因子) 全部:(脑源性神经营养因子) or 全部:(matrix metalloproteinase-9) or 全部:(动脉硬度) or 全部:(动脉僵硬度) or 全部:(踝肱指数) or 全部:(脉搏波速度)

#4: #1 AND #2 AND #3
